# Shadow of diabetes over cardiovascular disease: comparative quantification of population-attributable all-cause and cardiovascular mortality

**DOI:** 10.1186/1475-2840-11-69

**Published:** 2012-06-15

**Authors:** Mohammadreza Bozorgmanesh, Farzad Hadaegh, Farhad Sheikholeslami, Arash Ghanbarian, Fereidoun Azizi

**Affiliations:** 1 Prevention of Metabolic Disorders Research Center, Research Institute for Endocrine Sciences (RIES) Shahid Beheshti University of Medical Science, Tehran, Iran; 2 Endocrine Research Center, Research Institute for Endocrine Sciences (RIES) Shahid Beheshti University of Medical Sciences, Tehran, Iran

**Keywords:** Cardiovascular disease, Diabetes, Mortality, Population-attributable mortality

## Abstract

**Background:**

We contrasted impacts on all-cause and cardiovascular disease (CVD) mortality of diabetes vs. CVD.

**Methods:**

Among participants the Tehran lipid and glucose study aged ≥ 30 years (n = 9752), we selected those who participated in the follow-up study until 20 March 2009 (n = 8795). Complete data on covariate were available for 8, 469 participants, contributing to a 67935 person-year follow up. In the analysis of outcomes (all-cause and CVD mortality), diabetes and CVD were assessed using Cox proportional hazard regression model adjusting for established CVD risk factors. We used population attributable hazard fraction (PAHF) and rate advancement period (RAP) that expresses how much sooner a given mortality rate is reached among exposed than among unexposed individuals.

**Results:**

Ten percent of the participants self-reported to have pervious CVD, and diabetes was ascertained in 17% of participants at baseline examination. During a median follow-up of 9 years 386 participants died of which 184 were due to CVD. All-cause and CVD mortality rate (95% CIs) were 5.5 (5.0-6.1) and 2.6 (2.3-3.0) per 1000 person-year, respectively. The PAHF of all-cause mortality for diabetes 9.2 (7.3-11.1) was greater than the one for CVD 3.5 (1.1-5.5). RAP estimates for all-cause mortality associated with diabetes ranged from 7.4 to 8.6 years whereas the RAP estimates for all-cause mortality associated with CVD ranged from 3.1 to 4.3 years. The PAHF of CVD mortality for diabetes 9.4 (6.8-12.0) was greater than the one for CVD 4.5 (1.8-7.0). RAP estimates for CVD mortality associated with diabetes ranged from 8.2 to 9.8 years whereas the RAP estimates for CVD mortality associated with CVD ranged from 4.7 to 6.7 years.

**Conclusions:**

We demonstrated that diabetes, which was shown to be keeping pace with *prevalent* CVD in terms of conferring excess risk of *incident* CVD, is currently causing more deaths in the population than does CVD.

## **Background**

Mortality is an important measure of population health and is often used to assign priorities in health interventions [1,2]. Cardiovascular disease (CVD) has long been known as the leading cause of mortality and morbidity in developed countries [3]. The recognition and management of cardiovascular risk factors led to a decline in CVD related mortality rates in the 1960s. However, the increases in the prevalence of type 2 diabetes mellitus (hereafter diabetes) and obesity are potential warning signs that the hard-fought gains in mortality improvements might be arrested or even reversed [4,5]. The World Health Report 1999 estimates that in 1998, 78% of the burden of non-communicable diseases and 85% of the CVD burden arose from the low and middle income countries [6]. The high burdens of CVD in the developing countries are attributable to the increasing incidence of atherosclerotic diseases, perhaps due to urbanization [7-9]. Diabetes is not only a current common disease but its prevalence is expected to increase, especially in developing countries [10,11]. The target organ damage has been observed to increase the risk of cardiovascular complications independently of the existing estimated risk [12].

Diabetes has been shown by some studies to be a CVD equivalent in contribution to the CVD mortality in some studies [13-17]. These studies have solely relied on relative risk, while neglecting the prevalence of the diabetes and CVD. Furthermore, diabetes and CVD have never been weighted against each other with respect to the all-cause mortality. Routinely reported statistics based on death certification seriously underestimate mortality from diabetes. In fact, mortality from diabetes is underestimated four- to fivefold by methods of analysis of death certification data which use only underlying cause of death [13]. Individuals with diabetes most often die of cardiovascular and renal disease and not from a cause uniquely related to diabetes, such as ketoacidosis or hypoglycemia [14]. Therefore, mortality attributable to diabetes could be expected to be much higher, since diabetes is a serious and chronic condition. Although some developed countries have documented an improved survival of persons with diabetes, the increased prevalence is most likely due to increased incidence rather than improved survival [15].

Population risks of CVD and all-cause mortality due to diabetes have been previously documented in different population [16,17]. It has also been shown that population-attributable risk fraction should be periodically recalculated so that we can adequately capture trends in the population [18]. As the issue is of considerable clinical importance, particularly for public health strategies aimed at reducing mortality, we contrasted the population-wide impacts on the all-cause and CVD mortality of diabetes vs. CVD. We used population attributable hazard fraction (PAHF) as an epidemiologic tool which takes into account the prevalence of risk factors as well as the strength of their associations with mortality and guides policymakers in planning public health interventions priorities [19].

## **Methods**

### **Study population**

Detailed descriptions of the Tehran lipid and glucose study (TLGS) have been reported elsewhere [20,21]; in brief, the TLGS is a large scale, long term, community-based prospective study performed on a representative sample of residents of district No. 13 of Tehran, capital of Iran. Age and sex distributions of the population in the district were representative of the overall population of Tehran at the time of the baseline examination. The TLGS, has two major components: a cross-sectional prevalence study of non-communicable disease and associated risk factors, implemented between March 1999 and December 2001, and a prospective follow-up study. Data collection is ongoing, designed to continue for at least 20 years, at 3-year intervals. A total of 27 340 residents aged ≥3 years were invited by telephone call, of which 15, 010 residents participated in first examination cycle and another 3 ,551 residents were first examined at the second examination cycle. Participants were categorized into the cohort (n = 10 394) and intervention groups (n = 8 167), the latter to be educated for implementation of life style modifications. For the current study, among participants aged ≥30 years (n = 9 752), we selected those who participated in the follow-up study until 20 March 2009 (n = 8, 795). Complete data on covariate were available for 8, 469 participants, contributing to a 67, 935 person-year follow up. At the time of this study, the median follow up time was 9 years.

### **Clinical and laboratory measurements**

A trained interviewer collected information using a pretested questionnaire. The information obtained included demographic data, drug history, past medical history of CVD, hypertension, and diabetes and smoking status [22]. After a 15-minute rest in the sitting position, two measurements of blood pressure were taken, on the right arm, using a standardized mercury sphygmomanometer (calibrated by the Iranian Institute of Standards and Industrial Researches); the mean of the two measurements was considered as the participant’s blood pressure.

A blood sample was drawn between 7:00 and 9:00 AM from all study participants, after 12 to 14 hours overnight fasting. All the blood analyses were undertaken at the TLGS research laboratory on the day of blood collection. Plasma glucose was measured using an enzymatic colorimetric method with glucose oxidase. Fasting plasma glucose (FPG) measurement was performed for all participants, and the standard 2-hour post-challenge plasma glucose (2 h-PCPG) test for those not on glucose-lowering drugs. Total cholesterol (TC) was assayed, using the enzymatic colorimetric method with cholesterol esterase and cholesterol oxidase. High-density lipoprotein cholesterol (HDL-C) was measured after precipitation of the apolipoprotein B containing lipoproteins with phosphotungistic acid. Analyses were performed using Pars Azmon kits (Pars Azmon Inc., Tehran, Iran) and a Selectra 2 auto-analyzer (Vital Scientific, Spankeren, Netherlands). All samples were analyzed when internal quality control met the acceptable criteria. The intra and inter-assay coefficients of variation were both <2.2% for plasma glucose, and 0.5 and 2% for TC, respectively [20].

### **Outcome measurements**

Details of cardiovascular outcomes have been published elsewhere [23]. In this ongoing study every TLGS’ participant is followed up for any medical event during the previous year, by telephone. They are questioned by a trained nurse regarding any medical conditions or whether a related event have occurred, a trained physician collects complementary data during a home visit and/or a visit to the respective hospital to collect data from the participants medical files. In the case of mortality, data are collected from the hospital or the death certificate by an authorized local physician. Collected data are evaluated by an outcome committee consisting of a principal investigator, an internist, an endocrinologist, a cardiologist, an epidemiologist, and the physician who collects the outcome data. Other experts are invited for evaluation of non-communicable disorders, as needed. A specific outcome for each event is assigned according to International Statistical Classification of Diseases and Related Health Problems criteria, 10th Revision, and American Heart Association classification for cardiovascular events [20,24,25]. Coronary heart disease (CHD) includes cases of definite myocardial infarction (MI) diagnosed by electrocardiogram (ECG) and biomarkers, probable MI (positive ECG findings plus cardiac symptoms or signs and biomarkers showing negative or equivocal results), unstable angina pectoris (new cardiac symptoms or changing symptom patterns and positive ECG findings with normal biomarkers), angiographic proven CHD and CHD death. CVD is specified as a composite measure of any CHD events, stroke, or cerebrovascular death.

### **Definition of terms**

Current smoker was defined as a person who smokes cigarettes daily or occasionally. A previous history of CVD reflected any prior diagnosis of CVD by a physician. In accordance with the definition provided by American Diabetes Association, participants were classified as having diabetes at the baseline if they met at least one of these criteria: FPG ≥7 mmol.l^-1^, or 2 h-PCPG ≥ 11.1 mmol.l^-1^ or taking anti-diabetic medication [16].

### **Statistics analysis**

Findings on covariate variables are expressed as means (SD) or percentages for continuously distributed and categorical variables, respectively.

In the analysis of outcomes (all-cause and CVD mortality), diabetes and CVD were assessed using Cox proportional hazard regression model. Survival time was the time from start of the follow-up period to the date of the first incident, CVD event or death (failure). The censoring time of an individual was the time from entry into the study to loss to follow-up or the end of the study (20^th^ March 2009), whichever happened first. Censored observation meant the individuals either refused to participate further in the study (lost to follow-up), died (from non-CVD causes), when death was not the study outcome (competing risk) or continued until 20^th^ March 2009 when the study was ended (administrative censoring). Potential confounding effects of age, smoking, systolic blood pressure, use of anti-hypertensive drugs, lifestyle modification intervention, total and HDL cholesterol were accounted for in the multivariate regression models. We also examined if direction or magnitude of the association of diabetes and CVD with different endpoints were modified by intervention, or each other. As such, the interaction term for intervention × diabetes, intervention × CVD, and diabetes × CVD were introduced into regression models. The significance of interactions (lack of independence) was tested by likelihood ratio test.

Wald tests of the linear hypotheses concerning the Cox regression models coefficients (paired homogeneity test) were performed to test the null hypotheses that the hazard ratios (effect size) for diabetes were equal to those for CVD.

Recently, Chen et al. and Samuelsen et al. have proposed a definition for population–attributable fraction (PAF) for cohort studies with time-to-event, i.e. Population Attributable Hazard Fraction (PAHF). The PAHF is defined based on the effect of the hypothetical risk factor modification to the low-risk level; it is estimated at the instantaneous time point t:

(1) $$PAHF=\frac{h(t)-h_{1}(t)}{h(t)}=\frac{p(t)(HR\left(t\right)-1)}{1+p(t)(HR\left(t\right)-1)}\text{,}$$

where *p* is proportion exposed at time *t* and HR(*t*) = *h*_*2*_*(t)/h*_*1*_*(t)* denotes instantaneous hazard ratio at time *t*[26,27]. This measure describes the approximate proportion of events that could be avoided by the risk factor modification in a short time interval *t, t +* Δ*t*, where Δt *→* 0. We used proportion exposed at baseline, *p = p*(0) as suggested [27,28]. As such the formula corresponds to the traditional PAF [29] formula as was presented in the literatures by Levin [19].

Rate advancement period (RAP) has been encouraged to be used as an effective risk communication tool. It expresses how much sooner a given mortality rate is reached among exposed than among unexposed individuals [30]. RAPs for diabetes and CVD were calculated in years as suggested by literature [31].

### **Participants and adjustment for potential selection bias**

Baseline age, use of anti-hypertensive drugs, systolic blood pressure, smoking, diabetes, and history of CVD, were included in the logistic model with participation as outcome. The probability of participation was estimated using logistic model and used as a propensity score. We added this propensity score to the survival models as a covariate and examined if the probability of participation was associated with incident CVD or mortality. The probability of participation was not associated with any of the outcomes under the investigation (P values > 0.8) and the parameter estimates remained essentially unchanged. Therefore, the selection bias is unlikely to have affected our estimations.

We set the statistical significance level at a two-tailed type I error of 0.05. All statistical analyses were performed using STATA version 12 (STATA, College Station, Texas USA).

### **Medical ethics**

We certify that all applicable institutional and governmental regulations concerning the ethical use of human volunteers were followed during this research. Informed written consent was obtained from all participants and the Ethical Committee of the Research Institute for Endocrine Sciences approved this study.

## **Results**

Baseline characteristics of the participants are presented in Table 1. Mean age of the participants was 33.3 years (19.0). Ten percent of the participants self-reported to have pervious CVD, and diabetes was ascertained in 17% of participants at baseline examination. During a median follow-up of 9 years 386 participants died of which 184 were due to CVD. All-cause and CVD mortality rate (95% CIs) were 5.5 (5.0-6.1) and 2.6 (2.3-3.0) per 1000 person-year, respectively.

**Table 1:** Characteristics of the participants

	**Women**	**Men**	**Total**
Observations (n)	4 762	4 033	8 795
Age (year)	33.24 (18.23)	33.34 (19.97)	33.29 (19.01)
Systolic blood pressure (mmHg)	114.62 (19.41)	115.88 (18.32)	115.17 (18.95)
Total cholesterol (mmol.l^-1^)	5.2 (1.26)	4.95 (1.13)	5.09 (1.21)
High-density lipoprotein Cholesterol (mmol.l^-1^)	1.16 (0.28)	1.04 (0.26)	1.11 (0.28)
Antihypertensive drug (users vs. non-users)	978 (0.21)	466 (0.12)	1 444 (0.16)
Current Smoking (smokers vs. non-smokers)	185 (0.04)	1 151 (0.30)	1 336 (0.15)
History of cardiovascular disease (yes vs. no)	373 (0.9)	542 (0.11)	915 (0.10)
Diabetes (yes vs. no)	631 (0.16)	818 (0.18)	1 449 (0.17)
Cardiovascular mortality (per 1000 person-year)	1.5 (1.1-1.9)	4.0 (3.4-4.8)	2.6 (2.3-3.0)
All-cause mortality (per 1000 person-year)	3.6 (3.1-403)	7.8 (6.9-8.8)	5.5 (5.0-6.1)

Among women, effects of diabetes and CVD on all-cause and CVD mortality were independent of each other. However, among men diabetes and CVD mutually modified effects of each other. Therefore, we excluded participants with both diabetes and CVD; as such we compared participants with diabetes/without CVD with those with CVD/without diabetes. Nonetheless, the point estimates for PAHFs and RAP remained essentially unchanged. To avoid complexity, thus, we reported results obtained from original sample assuming that diabetes and CVD independently contributed to the all-cause and CVD mortality (data not shown). The intervention did not contributed to the incidence of the outcomes under the investigation, neither did it modify the effects of CVD or diabetes on the outcomes (P values > 0.2).

Table 2 presents the comparative quantification of CVD and all-cause mortality attributable to diabetes and history of previous CVD. The HRs for all-cause mortality of diabetes was greater than those of CVD. The difference, however, did not achieve statistical significance among men. The PAHF of all-cause mortality for diabetes 9.2 (7.3-11.1) was greater than the one for CVD 3.5 (1.1-5.5). RAP estimates for all-cause mortality associated with diabetes ranged from 7.4 to 8.6 years whereas the RAP estimates for all-cause mortality associated with CVD ranged from 3.1 to 4.3 years.

**Table 2:** Effects of diabetes and history of cardiovascular disease on mortality from all-cause or cardiovascular disease

	**Female**		**Male**		**Total**	
	**HR (95% CIs)**	**P value**	**HR (95% CIs)**	**P value**	**HR (95% CIs)**	**P value**
All-cause mortality						
History of cardiovascular disease	1.47 (0.98-2.20)	0.061	1.47 (1.03-2.11)	0.034	1.48 (1.15-1.91)	0.002
Diabetes	2.66 (1.83-3.85)	0.000	1.81 (1.33-2.46)	0.000	2.06 (1.65-2.57)	0.000
Cardiovascular mortality						
History of cardiovascular disease	1.62 (0.92-2.87)	0.096	1.70 (1.08-2.68)	0.022	1.61 (1.13-2.29)	0.008
Diabetes	2.76 (1.58-4.80)	0.000	1.93 (1.28-2.90)	0.002	2.17 (1.57-3.01)	0.000

Models were adjusted for age, sex (were not sex-specific), current smoking, systolic blood pressure, using antihypertensive drugs, total and high-density lipoprotein cholesterol.

Tables 3 and 4 presents the comparative quantification of CVD and all-cause and CVD mortality attributable to diabetes and history of previous CVD. The HRs for CVD mortality of diabetes was greater than those of CVD. The difference, however, did not achieve statistical significance. The PAHF of CVD mortality for diabetes 9.4 (6.8-12.0) was greater than the one for CVD 4.5 (1.8-7.0). RAP estimates for CVD mortality associated with diabetes ranged from 8.2 to 9.8 years whereas the RAP estimates for CVD mortality associated with CVD ranged from 4.7 to 6.7 years.

**Table 3:** Comparing burden of all-cause mortality due to diabetes with those due to CVD

	**Diabetes**	**History of CVD**
Women		
Prevalence (%)	17.7	11.4
HR^1^ (95%CIs)	2.66 (1.83-3.85)	1.47 (0.98-2.20)
Wald χ^2^	4.65 (P for paired homogeneity test^2^ = 0.031 )	
PAHF^3^ (95%CIs)	11.0 (8.4-13.5)	3.7 (0.0-7.3)
RAP^4^ (years)	8.6	3.1
Men		
Prevalence (%)	16.3	9.3
HR^1^ (95%CIs)	1.81 (1.33-2.46)	1.47 (1.03-2.11)
Wald χ^2^	1.19 (P for paired homogeneity test^2^ = 0.275 )	
PAHF^3^ (95%CIs)	7.9 (5.3-10.5)	3.0 (0.01-5.7)
RAP^4^ (years)	7.4	4.3
Total		
Prevalence (%)	17.1	10.4
HR^1^ (95%CIs)	2.06 (1.65-2.57)	1.48 (1.15-1.91)
Wald χ^2^	5.26 (P for paired homogeneity test^2^ = 0.022 )	
PAHF^3^ (95%CIs)	9.2 (7.3-11.1)	3.3 (1.1-5.5)
RAP^4^ (years)	8.0	3.5

CVD, cardiovascular disease; HR, hazard ratio; PAHF, population-attributable hazard fraction; RAP, rate advancement period.

1. HRs and their 95% CIs were estimated by implementing the survival proportional hazard (Cox) regression analysis.

2. Wald tests of the linear hypotheses concerning the Cox survival regression models coefficients (paired homogeneity test) were performed to test the null hypotheses that the hazard ratios (effect size) for prevalent diabetes were equal to those for prevalent CVD [32].

$PAHF=\frac{h(t)-h_{1}(t)}{h(t)}=\frac{p(t)(HR\left(t\right)-1)}{1+p(t)(HR\left(t\right)-1)}\text{,}$ where p(*t*) is the proportion exposed to hypertension at time *t*. We used proportion exposed at baseline, *p* = *p*(0).

4. RAP expresses how much sooner a given mortality rate is reached among exposed than among unexposed individuals [30].

**Table 4:** Comparing burden of CVD mortality due to diabetes with those due to CVD

	**Diabetes**	**History of CVD**
Women		
Prevalence (%)	13.9	8.7
HR^1^ (95%CIs)	2.76 (1.58-4.80)	1.62 (0.92-2.87)
Wald χ^2^	1.69 (P for paired homogeneity test^2^ = 0.194 )	
PAHF^3^ (95%CIs)	11.5 (7.8-15.0)	4.9 (0.3-9.4)
RAP^4^ (years)	9.8	4.7
Men		
Prevalence (%)	14.1	6.7
HR^1^ (95%CIs)	1.92 (1.28-2.90)	1.70 (1.08-2.68)
Wald χ^2^	0.14 (P for paired homogeneity test^2^ = 0.711 )	
PAHF^3^ (95%CIs)	8.0 (4.4-11.5)	4.3 (1.5-7.1)
RAP^4^ (years)	8.2	6.7
Total		
Prevalence (%)	13.5	6.4
HR^1^ (95%CIs)	2.17 (1.57-3.01)	1.61 (1.13-2.29)
Wald χ^2^	1.38 (P for paired homogeneity test^2^ = 0.239 )	
PAHF^3^ (95%CIs)	9.4 (6.8-12.0)	4.5 (1.8-7.0)
RAP^4^ (years)	9.0	5.5

CVD, cardiovascular disease; HR, hazard ratio; PAHF, population-attributable hazard fraction; RAP, rate advancement period.

1. HRs and their 95% CIs were estimated by implementing the survival proportional hazard (Cox) regression analysis.

2. Wald tests of the linear hypotheses concerning the Cox survival regression models coefficients (paired homogeneity test) were performed to test the null hypotheses that the hazard ratios (effect size) for prevalent diabetes were equal to those for prevalent CVD [32].

$PAHF=\frac{h(t)-h_{1}(t)}{h(t)}=\frac{p(t)(HR\left(t\right)-1)}{1+p(t)(HR\left(t\right)-1)}\text{,}$ where p(*t*) is the proportion exposed to hypertension at time *t*. We used proportion exposed at baseline, *p* = *p*(0).

4. RAP expresses how much sooner a given mortality rate is reached among exposed than among unexposed individuals [30].

## **Discussion**

Using data from a well-characterized prospective cohort study of men and women, we comparatively quantified the population-wide burden of all-cause and CVD mortality attributable to diabetes and CVD. We observed that population mortality conferred by diabetes is not lower than those conferred by previous CVD events. In fact, among women population mortality from any cause conferred by diabetes was significantly higher than those conferred by previous CVD. We also applied the concept of RAP that provides an excellent translation into lay language. Accordingly, we observed that diabetic patients died 7–10 years sooner than did those without diabetes and that patients with previous CVD died 3–7 years sooner than did those without CVD.

The PAHF, which incorporates both risk factor prevalence and its predictive value (i.e. relative or excess risk), provide a measure of effectiveness that can be broadly helpful in deciding which risk factor should be targeted by health interventions [7]. Our finding shows that mortality could be reduced twice as much by elimination from the population of diabetes as by elimination from the population of CVD.

Beverly Levine [33] argued that the most illuminating questions about the attributable fraction are: What interventions are available to cause the assumed reduction in risk among the exposed and the consequent estimated reduction in disease burden? What proportion of disease risk could be eliminated if the absolute risk in the exposed were to suddenly and sustainably go to the level of absolute risk in the unexposed, while nothing else, including absolute risk in the unexposed, were to change?

While there is no doubt that diabetes harbors a causal effect on CVD [34-38] and all-cause mortality [36,39,40], whether these effects are exerted through hyperglycemia or whether it is just an innocent bystander has been increasingly challenged [41-43]. Overt diabetes has been shown to modify the effect on mortality of hyperglycemia [44]. Insulin resistance another feature of diabetes, has been shown to increase the risk of new CVD events [45]. It has been shown that glucose-related risks do exist before levels currently used to define diabetes are reached [46,47]. Intensive glucose lowering has long been known to decrease microvascular complications of diabetes. The ADVANCE study (Action in Diabetes and Vascular Disease: Preterax and Diamicron Modified Release Controlled Evaluation) has sprinkled some hope that intensive glucose control may confer some benefit on CVD [2]. In the Action to Control Cardiovascular Risk in Diabetes (ACCORD) study increased mortality has been attributed to failing to achieve targets [48]. Traditionally, intensive glucose lowering was not achieved without hypoglycemia [8], weight gain [49], and increased cardiovascular risk (as a side effect of glucose lowering agents ) [2]. It was therefore associated with an increased CVD and all-mortality [4]. Recently, however, there has been an ever-expanding body of evidences on the effectiveness of new approaches to intensify glucose control. New approaches have been shown to reverse diabetes [50], have lower risk of hypoglycemia [51], promote weight loss, and confer decreased CVD risk among diabetic patients. Findings of the current study can, in a population-wide scale, determine the potential borders of the achievement for novel approaches to treat diabetes. Novel therapies can help reduce glucose levels much safer to much lower levels (which would be concomitant reduction in mortality from microvascular complications) meanwhile diabetes-specific modification of atherosclerosis may reduce CVD mortality among diabetic patients. Therefore novel glucose lowering agent may confer a potential for decrease in all-cause mortality. Furthermore, type 2 diabetes has been shown to be prevented or the onset delayed by lifestyle modification, which although involves costs, imposes no harms [52,53].

We are aware that diabetes and CVD are correlated and their effects on outcomes are not mutually exclusive but rather mutually modifiable. Nonetheless, the likelihood ratio test revealed no evidence of significant diabetes × CVD interaction among women. Among men, where interactions were statistically significant, we excluded data on participants with both diabetes and CVD. This subgroup analysis allowed us to directly compare men who had diabetes/without CVD with those who had CVD/without diabetes. We observed that relative importance of the two conditions remained unchanged; our interpretation is, thus, unlikely to have been considerably affected by the effect-modification. Furthermore, allowing for the CVD-modified effects of diabetes simply mean to calculate PAHFs for individuals with diabetes who also had CVD. While it could be theoretically appealing, practically, it is not currently recommended to intensify glucose control among diabetic patients with established macrovascular complication in order to prevent cardiovascular disease [54]. New pharmacologic approaches are on the horizon that offer some hope, though [55].

The clinical implications of the findings reported herein lies in its observable practical consequences. Diabetic patients have residual risk or CVD mortality even after treatment of established CVD risk factors like blood pressure and lipid levels [56]. Approval of glucose-lowering agents may no longer be prudent unless they offer additional beneficial impact on CVD or mortality. Such therapeutic approaches in the light of the current findings will have much potential to improve the population longevity. We await more clinical trials to see if such therapeutic approaches live up to our expectations. The findings of the current study have some public health implications as it allows clinicians more effectively communicate the diabetes-associated risk their patients. For example, a RAP of 8.6 years can be used to explain to a 50-year-old diabetic woman that she has the same risk of death as does a 59-year-old non-diabetic woman.

### **Study strengths and limitations**

#### **Strength**

Strength of the present study lies in its prospective nature, the use of a large population-based-cohort of both sexes, accurate and valid data on risk factors at baseline, continuous surveillance of mortality and CVD events based on standard criteria. We used standard methods for measurement of plasma glucose including 2-PCPG. The fact that we adjusted for established CVD risk factors (age, systolic blood pressure, total and HDL cholesterol, cigarette smoking, and use of antihypertensive drugs) makes it unlikely that the strong associations of the all-cause or CVD mortality to diabetes observed here are due to confounding with known risk factors, although in an observational study that possibility can never be definitely excluded. The practical usefulness of RAP in terms of risk communication still waits thorough evaluation. However, its simple calculation and intuitive appeal should make it a worthwhile endeavor [31].

#### **Limitations**

Some limitations to our study merit mentioning.

1. The number of incident events precluded stratification of analyses by age.

2. Our findings may not apply to individuals of different racial and socioeconomic groups than those in the TLGS population (entirely Persian middle class).

3. We do not have sufficient data to accurately assess the relations of various cause-specific deaths to diabetes. Using aggregate clinical outcome could have potentially attenuated the strength of the associations.

4. Some participants might have developed diabetes or non-fatal CVD during follow up. This misclassification renders our results to regression dilution. Regression dilution, however, biases the estimates towards the null and, thus, is unlikely to have significant affected the results obtained and the interpretations inferred herein.

5. Finally, we only considered glycemic status once intra-individual variability in these measures may have misclassified participants, but this problem would produce an underestimate of the effects of glycemia on outcomes.

## **Conclusions**

We have comparatively quantified the public health impact of diabetes and history of previous CVD in terms of all-cause and CVD mortality. We demonstrated that diabetes, which was shown to be keeping pace with *previous* CVD events in terms of conferring excess risk of *incident future* CVD events, is currently causing more deaths in the population than do *previous* CVD events

## **Abbreviations**

CHD: Coronary heart disease; CVD: Cardiovascular disease; ECG: Electrocardiography; FPG: Fasting plasma glucose; HDL-C: High-density lipoprotein cholesterol; MI: Myocardial infarction; PAHF: Population-attributable hazard function; PCPG: Post-challenge plasma glucose; RAP: Rate advancement period; TC: Total cholesterol; TLGS: Tehran lipid and glucose study.

## **Competing interests**

The authors declare that they have no competing interests.

## **Authors’ contributions**

MB conceived of and designed the study, carried out the statistical analysis, interpreted the results, and drafted the manuscript. FH, and FA helped critically revised the manuscript. All authors read and approved the final manuscript.

## **Financial disclosure**

This study was supported by grant No. 121 from the National Research Council of the Islamic Republic of Iran. The funders had no role in study design, data collection and analysis, decision to publish, or preparation of the manuscript.

## References

[B1] Tuomilehto J Rastenyte D Birkenhäger WH Thijs L Antikainen R Bulpitt CJ Fletcher AE Forette F Goldhaber A Palatini P Effects of Calcium-Channel Blockade in Older Patients with Diabetes and Systolic Hypertension New England Journal of Medicine 1999 340 677 684 10.1056/NEJM199903043400902 10053176

[B2] Patel A MacMahon S Chalmers J Neal B Billot L Woodward M Marre M Cooper M Glasziou P Grobbee D Intensive blood glucose control and vascular outcomes in patients with type 2 diabetes N Engl J Med 2008 358 2560 2572 18539916 10.1056/NEJMoa0802987

[B3] Edejer TT-T Disseminating health information in developing countries: the role of the internet BMJ 2000 321 797 800 10.1136/bmj.321.7264.797 11009519 PMC1118616

[B4] Hemmingsen B Lund SS Gluud C Vaag A Almdal T Hemmingsen C Wetterslev J Intensive glycaemic control for patients with type 2 diabetes: systematic review with meta-analysis and trial sequential analysis of randomised clinical trials BMJ 2011 343 d6898 10.1136/bmj.d6898 22115901 PMC3223424

[B5] Chen G McAlister FA Walker RL Hemmelgarn BR Campbell NRC Cardiovascular Outcomes in Framingham Participants With Diabetes Hypertension 2011 57 891 897 10.1161/HYPERTENSIONAHA.110.162446 21403089 PMC3785072

[B6] Haffner SM Lehto S Rönnemaa T Pyörälä K Laakso M Mortality from Coronary Heart Disease in Subjects with Type 2 Diabetes and in Nondiabetic Subjects with and without Prior Myocardial Infarction New England Journal of Medicine 1998 339 229 234 10.1056/NEJM199807233390404 9673301

[B7] Ghosh A Castle PE Invited commentary: the importance of prevalence in the effectiveness of a (bio)marker Am J Epidemiol 2011 173 1388 1390 discussion 1391 10.1093/aje/kwr016 21571873 PMC3145395

[B8] Zoungas S Patel A Chalmers J de Galan BE Li Q Billot L Woodward M Ninomiya T Neal B MacMahon S Severe Hypoglycemia and Risks of Vascular Events and Death New England Journal of Medicine 2010 363 1410 1418 10.1056/NEJMoa1003795 20925543

[B9] Wang WS Wahlqvist ML Hsu CC Chang HY Chang WC Chen CC Age-and gender-specific population attributable risks of metabolic disorders on all-cause and cardiovascular mortality in Taiwan BMC Public Health 2012 12 111 10.1186/1471-2458-12-111 22321049 PMC3305485

[B10] Harati H Hadaegh F Saadat N Azizi F Population-based incidence of Type 2 diabetes and its associated risk factors: results from a six-year cohort study in Iran BMC Public Health 2009 9 186 10.1186/1471-2458-9-186 19531260 PMC2708154

[B11] Hadaegh F Bozorgmanesh MR Ghasemi A Harati H Saadat N Azizi F High prevalence of undiagnosed diabetes and abnormal glucose tolerance in the Iranian urban population: Tehran Lipid and Glucose Study BMC Public Health 2008 8 176 10.1186/1471-2458-8-176 18501007 PMC2413226

[B12] Gomez-Marcos MA Recio-Rodríguez JI Patino-Alonso MC Agudo-Conde C Gomez-Sanchez L Rodriguez-Sanchez E Gomez-Sanchez M Garcia-Ortiz L Yearly evolution of organ damage markers in diabetes or metabolic syndrome: data from the LOD-DIABETES study Cardiovascular Diabetology 2011 10 90 10.1186/1475-2840-10-90 21999369 PMC3214163

[B13] Westman M Etzion D Danon E Job insecurity and crossover of burnout in married couples Journal of Organizational Behavior 2001 22 467 481 10.1002/job.91

[B14] Nash ES Teenage pregnancy-need a child bear a child South African Medical Journal 1990 77 147 151 2406956

[B15] Trussell J Teenage pregnancy in the United States Family Planning Perspectives 1988 20 262 272 10.2307/2135482 3229472

[B16] Bozorgmanesh M Hadaegh F Sheikholeslami F Azizi F Cardiovascular risk and all cause mortality attributable to diabetes: Tehran Lipid and Glucose Study J Endocrinol Invest 2011 35 14 20 21586894 10.3275/7728

[B17] Saito I Kokubo Y Yamagishi K Iso H Inoue M Tsugane S Diabetes and the risk of coronary heart disease in the general Japanese population: The Japan Public Health Center-based prospective (JPHC) study Atherosclerosis 2011 216 187 191 10.1016/j.atherosclerosis.2011.01.021 21300356

[B18] Grau M Subirana I Elosua R Fitó M Covas MI Sala J Masiá R Ramos R Solanas P Cordon F Why should population attributable fractions be periodically recalculated?: An example from cardiovascular risk estimation in southern Europe Preventive medicine 2010 51 78 84 10.1016/j.ypmed.2010.03.012 20362610

[B19] Kirby D Emerging answers 2007: Research findings on programs to reduce teen pregnancy and sexually transmitted diseases National Campaign to Prevent Teen and Unplanned Pregnancy. Retrieved February 2007 23 2009

[B20] Azizi F Ghanbarian A Momenan AA Hadaegh F Mirmiran P Hedayati M Mehrabi Y Zahedi-Asl S Prevention of non-communicable disease in a population in nutrition transition: Tehran Lipid and Glucose Study phase II Trials 2009 10 5 10.1186/1745-6215-10-5 19166627 PMC2656492

[B21] Bozorgmanesh M Hadaegh F Khalili D Azizi F Prognostic significance of the Complex "Visceral Adiposity Index" vs. simple anthropometric measures: Tehran Lipid and Glucose Study Cardiovascular Diabetology 2012 11 20 10.1186/1475-2840-11-20 22394430 PMC3376032

[B22] Freedman DS Kahn HS Mei Z Grummer-Strawn LM Dietz WH Srinivasan SR Berenson GS Relation of body mass index and waist-to-height ratio to cardiovascular disease risk factors in children and adolescents: the Bogalusa Heart Study Am J Clin Nutr 2007 86 33 40 17616760 10.1093/ajcn/86.1.33

[B23] Hadaegh F Harati H Ghanbarian A Azizi F Association of total cholesterol versus other serum lipid parameters with the short-term prediction of cardiovascular outcomes: Tehran Lipid and Glucose Study Eur J Cardiovasc Prev Rehabil 2006 13 571 577 10.1097/01.hjr.0000216552.81882.ca 16874147

[B24] Gibbons RJ Abrams J Chatterjee K Daley J Deedwania PC Douglas JS Ferguson TB Fihn SD Fraker TD Gardin JM ACC/AHA 2002 Guideline Update for the Management of Patients With Chronic Stable Angina--Summary Article: A Report of the American College of Cardiology/American Heart Association Task Force on Practice Guidelines (Committee on the Management of Patients With Chronic Stable Angina) Circulation 2003 107 149 158 10.1161/01.CIR.0000047041.66447.29 12515758

[B25] Braunwald E Antman EM Beasley JW Califf RM Cheitlin MD Hochman JS Jones RH Kereiakes D Kupersmith J Levin TN ACC/AHA Guideline Update for the Management of Patients With Unstable Angina and Non-ST-Segment Elevation Myocardial Infarction--2002: Summary Article: A Report of the American College of Cardiology/American Heart Association Task Force on Practice Guidelines (Committee on the Management of Patients With Unstable Angina) Circulation 2002 106 1893 1900 10.1161/01.CIR.0000037106.76139.53 12356647

[B26] Chen YQ Hu C Wang Y Attributable risk function in the proportional hazards model for censored time-to-event Biostatistics 2006 7 515 10.1093/biostatistics/kxj023 16478758

[B27] Samuelsen SO Eide GE Attributable fractions with survival data Statistics in Medicine 2008 27 1447 1467 10.1002/sim.3022 17694507

[B28] Silverberg MJ Smith MW Chmiel JS Detels R Margolick JB Rinaldo CR O’Brien SJ Muñoz A Fraction of Cases of Acquired Immunodeficiency Syndrome Prevented by the Interactions of Identified Restriction Gene Variants American journal of epidemiology 2004 159 232 241 10.1093/aje/kwh036 14742283

[B29] Laaksonen MA Knekt P Härkänen T Virtala E Oja H Estimation of the Population Attributable Fraction for Mortality in a Cohort Study Using a Piecewise Constant Hazards Model American journal of epidemiology 2010 171 837 847 10.1093/aje/kwp457 20197386

[B30] Siadat SH Buyut VC Selamat H Measuring service quality in E-retailing using SERVQUAL model IEEE 2008 1 7

[B31] Liese AD Hense H-W Brenner H Löwel H Keil U Assessing the Impact of Classical Risk Factors on Myocardial Infarction by Rate Advancement Periods American Journal of Epidemiology 2000 152 884 888 10.1093/aje/152.9.884 11085401

[B32] Abbate LM Stevens J Schwartz TA Renner JB Helmick CG Jordan JM Anthropometric measures, body composition, body fat distribution, and knee osteoarthritis in women Obesity (Silver Spring) 2006 14 1274 1281 10.1038/oby.2006.145 16899809

[B33] Levine B Peer Reviewed: What Does the Population Attributable Fraction Mean? Preventing chronic disease 2007 4 A14 17173722 PMC1832135

[B34] Casiglia E Zanette G Mazza A Donadon V Donada C Pizziol A Tikhonoff V Palatini P Pessina AC Cardiovascular mortality in non-insulin-dependent diabetes mellitus. A controlled study among 683 diabetics and 683 age- and sex-matched normal subjects European Journal of Epidemiology 2000 16 677 684 10.1023/A:1007673123716 11078126

[B35] Smith NL Barzilay JI Kronmal R Lumley T Enquobahrie D Psaty BM New-Onset Diabetes and Risk of All-Cause and Cardiovascular Mortality Diabetes Care 2006 29 2012 2017 10.2337/dc06-0574 16936145

[B36] Greenland P Knoll MD Stamler J Neaton JD Dyer AR Garside DB Wilson PW Major Risk Factors as Antecedents of Fatal and Nonfatal Coronary Heart Disease Events JAMA: The Journal of the American Medical Association 2003 290 891 897 10.1001/jama.290.7.891 12928465

[B37] Haffner SM Lehto S Ronnemaa T Pyorala K Laakso M Mortality from coronary heart disease in subjects with type 2 diabetes and in nondiabetic subjects with and without prior myocardial infarction N Engl J Med 1998 339 229 234 10.1056/NEJM199807233390404 9673301

[B38] Folsom AR Szklo M Stevens J Liao F Smith R Eckfeldt JH A prospective study of coronary heart disease in relation to fasting insulin, glucose, and diabetes. The Atherosclerosis Risk in Communities (ARIC) Study Diabetes Care 1997 20 935 942 10.2337/diacare.20.6.935 9167103

[B39] Panzram G Mortality and survival in Type 2 (non-insulin-dependent) diabetes mellitus Diabetologia 1987 30 123 131 10.1007/BF00274216 3556287

[B40] Stengård J Tuomilehto J Pekkanen J Kivinen P Kaarsalo E Nissinen A Karvonen M Diabetes mellitus, impaired glucose tolerance and mortality among elderly men: The Finnish cohorts of the seven countries study Diabetologia 1992 35 760 765 1511803 10.1007/BF00429097

[B41] Buse JB Should Postprandial Glucose Be Routinely Measured and Treated to a Particular Target? No! Diabetes Care 2003 26 1615 1618 10.2337/diacare.26.5.1615 12716828

[B42] Leosdottir M Willenheimer R Persson M Nilsson PM The association between glucometabolic disturbances, traditional cardiovascular risk factors and self-rated health by age and gender: A cross-sectional analysis within the Malmö Preventive Project Cardiovascular Diabetology 2011 10 118 10.1186/1475-2840-10-118 22204568 PMC3270001

[B43] Nishimura R Nakagami T Sone H Ohashi Y Tajima N Relationship between hemoglobin A1c and cardiovascular disease in mild-to-moderate hypercholesterolemic Japanese individuals: subanalysis of a large-scale randomized controlled trial Cardiovascular Diabetology 2011 10 1 6 10.1186/1475-2840-10-1 21714932 PMC3150244

[B44] Liu Y Yang Y Zhu J Tan H Liang Y Li J Prognostic significance of hemoglobin A1c level in patients hospitalized with coronary artery disease. A systematic review and meta-analysis Cardiovascular Diabetology 2011 10 1 9 10.1186/1475-2840-10-1 22074110 PMC3225330

[B45] Verhagen SN Wassink AMJ van der Graaf Y Gorter PM Visseren FLJ Insulin resistance increases the occurrence of new cardiovascular events in patients with manifest arterial disease without known diabetes. The SMART study Cardiovascular Diabetology 2011 10 100 108 10.1186/1475-2840-10-100 22098712 PMC3268731

[B46] Yadegari H Bozorgmanesh M Hadaegh F Azizi F Non-linear contribution of glucose measures to cardiovascular diseases and mortality: Reclassifying the Framingham's risk categories: A decade follow-up from the Tehran lipid and glucose study International Journal of Cardiology 2012 Epub ahead of print 10.1016/j.ijcard.2012.04.053 22578948

[B47] Schinner S Füth R Kempf K Martin S Willenberg HS Schott M Dinh W Scherbaum WA Lankisch M A progressive increase in cardiovascular risk assessed by coronary angiography in non-diabetic patients at sub-diabetic glucose levels Significance 2011 8 11 10.1186/1475-2840-10-56 PMC3142488 21702911

[B48] Riddle MC Counterpoint: Intensive Glucose Control and Mortality in ACCORD—Still Looking for Clues Diabetes Care 2010 33 2722 10.2337/dc10-1658 21115778 PMC2992221

[B49] HOOGWERF BJ Does intensive therapy of type 2 diabetes help or harm? Seeking accord on ACCORD Cleveland Clinic Journal of Medicine 2008 75 729 737 10.3949/ccjm.75.10.729 18939389

[B50] Lim E Hollingsworth K Aribisala B Chen M Mathers J Taylor R Reversal of type 2 diabetes: normalisation of beta cell function in association with decreased pancreas and liver triacylglycerol Diabetologia 2011 54 2506 2514 10.1007/s00125-011-2204-7 21656330 PMC3168743

[B51] Fonseca V Baron M Shao Q Dejager S Sustained efficacy and reduced hypoglycemia during one year of treatment with vildagliptin added to insulin in patients with type 2 diabetes mellitus Hormone and metabolic research 2008 40 427 10.1055/s-2008-1058090 18401832

[B52] Tuomilehto J Lindstrom J Eriksson JG Valle TT Hamalainen H Ilanne-Parikka P Keinanen-Kiukaanniemi S Laakso M Louheranta A Rastas M Prevention of type 2 diabetes mellitus by changes in lifestyle among subjects with impaired glucose tolerance N Engl J Med 2001 344 1343 1350 10.1056/NEJM200105033441801 11333990

[B53] Harati H Hadaegh F Momenan AA Ghanei L Bozorgmanesh MR Ghanbarian A Mirmiran P Azizi F Reduction in incidence of type 2 diabetes by lifestyle intervention in a middle eastern community Am J Prev Med 2010 38 628 636 e621 10.1016/j.amepre.2010.03.003 20494239

[B54] Skyler JS Bergenstal R Bonow RO Buse J Deedwania P Gale EAM Howard BV Kirkman MS Kosiborod M Reaven P Intensive glycemic control and the prevention of cardiovascular events: implications of the ACCORD, ADVANCE, and VA Diabetes Trials Circulation 2009 119 351 357 10.1161/CIRCULATIONAHA.108.191305 19095622

[B55] Cohen A Horton ES Progress in the treatment of type 2 diabetes: new pharmacologic approaches to improve glycemic control Current Medical Research and Opinion 2007 23 905 917 10.1185/030079907X182068 17407648

[B56] Nesto RW Singh PP Diabetes and residual risk of coronary heart disease Nat Clin Pract End Met 2007 3 71 71 10.1038/ncpendmet0398 17237830

